# Noradrenaline transporter PET reflects neurotoxin-induced noradrenaline level decrease in the rat hippocampus

**DOI:** 10.1186/s13550-023-01032-y

**Published:** 2023-09-15

**Authors:** Takayuki Sakai, Saori Hattori, Aya Ogata, Takashi Yamada, Junichiro Abe, Hiroshi Ikenuma, Masanori Ichise, Masaaki Suzuki, Kengo Ito, Takashi Kato, Yasuyuki Kimura

**Affiliations:** 1 Department of Clinical and Experimental Neuroimaging, Center for Development of Advanced Medicine for Dementia, Research Institute, National Center for Geriatrics and Gerontology (NCGG), Obu, Aichi 474-8511 Japan; 2 Department of Pharmacy, Faculty of Pharmacy, Gifu University of Medical Science (GUMS), Kani, Japan

**Keywords:** Noradrenaline, Noradrenaline Transporter, [^11^C]MRB, DSP-4, Positron emission tomography, Brain, Rat

## Abstract

**Background:**

The neuropathological changes of early Alzheimer’s disease (AD) include neurodegenerative loss of noradrenaline neurons in the locus coeruleus with decreasing noradrenaline availability in their projection areas such as the hippocampus. This diminishing noradrenaline availability is thought to play an important role pathophysiologically in the development of cognitive impairment in AD, because noradrenaline is not only essential for maintaining cognitive functions such as memory, learning and attention, but also its anti-inflammatory action, where its lack is known to accelerate the progression of AD in the mouse model. Therefore, the availability of in vivo biomarkers of the integrity of noradrenaline neurons may be beneficial for furthering our understanding of the role played by the noradrenaline system in the progressive cognitive dysfunction seen in AD patients. In this study, we investigated if PET imaging of noradrenaline transporters can predict the level of noradrenaline in the brain. Our hypothesis was PET measured noradrenaline transporter densities could predict the level of noradrenaline concentrations in the rat hippocampus after lesioning of noradrenaline neurons in this region.

**Results:**

We chemically lesioned the hippocampus of rats (*n* = 15) by administering a neurotoxin, DSP-4, in order to selectively damage axonal terminals of noradrenergic neurons. These rats then underwent PET imaging of noradrenaline transporters using [^11^C]MRB ((*S,S*)-[^11^C]Methylreboxetine). To validate our hypothesis, postmortem studies of brain homogenates of these rats were performed to measure both noradrenaline transporter and noradrenaline concentrations. [^11^C]MRB PET showed decreased noradrenaline transporter densities in a DSP-4 dose-dependent manner in the hippocampus of these rats. In turn, these PET measured noradrenaline transporter densities correlated very well with in vitro measured noradrenaline concentrations as well as in vitro transporter densities.

**Conclusions:**

[^11^C]MRB PET may be used as an in vivo biomarker of noradrenaline concentrations in the hippocampus of the neurodegenerating brain. Further studies appear warranted to extend its applicability to AD studies.

**Supplementary Information:**

The online version contains supplementary material available at 10.1186/s13550-023-01032-y.

## Background

Alzheimer’s disease (AD) is the leading cause of dementia with enormous impacts on affected individuals, families, caregivers, society at large and the health care system. In spite of intensive ongoing worldwide efforts to combat this condition, however, there are currently no definitive treatment methods to prevent or halt this slowly progressive neurodegenerative disorder. Pathologically, two hallmarks of AD are the abnormal accumulation of extra-neuronal β-amyloid plaques and neurofibrillary tangles containing hyperphosphorylated microtubule binding protein tau, leading to neuronal degeneration of multiple neural systems and progressive brain atrophy [1]. One such system involved in AD, which is of our interest here, is the noradrenergic system. Clinically, the cardinal feature of AD includes cognitive impairments such as loss of memory, learning and attention, poor insight/judgment, emotional and personality alterations [2]. In the early stage of AD, neurodegenerative loss of noradrenergic neurons in the locus coeruleus leads to a decrease in noradrenaline availability in their projection areas, such as the hippocampus. This diminishing noradrenaline availability is thought to play an important role in the development of cognitive impairment in AD, because noradrenaline is not only essential for maintaining cognitive functions such as memory, learning and attention [3–5], but also its anti-inflammatory action, where its lack is known to accelerate the progression of AD in the mouse model [6]. Therefore, the availability of in vivo biomarkers of the integrity of noradrenaline system may be beneficial for furthering our understanding of the role played by the noradrenalin system in the progressive cognitive dysfunction seen in AD patients.

As an imaging tool to detect and measure in vivo biomarkers of AD, positron emission tomography (PET) has been widely used. For example, various PET ligands have been developed to image β-amyloid and tau protein aggregates. In this study, we investigated if PET imaging of noradrenaline transporters could be an indirect biomarker of the integrity of noradrenaline neurons and the availability of noradrenaline itself in the brain. Noradrenaline transporter is a monoamine transporter nearly exclusively expressed on noradrenergic neurons and its function is to transport synaptically released noradrenaline back into the presynaptic noradrenaline neuron [7]. When noradrenaline neurons are damaged with neurodegeneration, noradrenaline transporter densities decrease by the damage itself and also by the process of downregulation in the response to decreased noradrenaline levels in the synaptic clefts. Because there is no way to measure noradrenaline levels in the living human brain noninvasively, we focused on the density of noradrenaline transporter to evaluate the level of noradrenaline in the brain.

Noradrenaline transporter densities in the brain have been measured using PET with [^11^C]MRB (*S,S*)-[^11^C]Methylreboxetine and (*S,S*)-[^18^F]FMeNER-D_2_ as well as other PET noradrenaline transporter ligands in rats, monkeys and humans [8–14]. In those studies with coadministration of blocking agents, [^11^C]MRB has been shown to specifically bind to noradrenaline transporters in the brain. [^11^C]MRB has also been used to detect age related decline of noradrenaline transporter densities in healthy humans [15, 16].

The purpose of the study was to investigate that PET measured noradrenaline transporter densities could predict the level of noradrenaline concentrations in the rat hippocampus after lesioning of noradrenaline neurons in this region. To accomplish this goal, we chemically lesioned the hippocampus of rats by administering a neurotoxin, DSP-4, in order to selectively damage axonal terminals of noradrenergic neurons. These rats then underwent PET imaging of noradrenaline transporters using [^11^C]MRB [8]. To validate our hypothesis, postmortem studies of brain homogenates of these rats were performed to measure both noradrenaline transporter and noradrenaline concentrations in the brain.

## Methods

### Chemicals

(2*S*,3*S*)-Desethylreboxetine was purchased from ABX (Radeberg, Germany). Methanol was HPLC grade from Merck. Noradrenaline, 3,4-dihydroxybenzylamine, and isoproterenol were purchased from Merck, citric acid monohydrate and sodium acetate from Kanto Chemical (Tokyo, Japan), ethylenediaminetetraacetic acid from Dojindo (Kumamoto, Japan), and sodium octane sulfonate from FUJIFILM Wako Pure Chemical (Osaka, Japan). All other chemicals were of analytical grade and were used without further pretreatment. Stock solutions of noradrenaline, 3,4-dihydroxybenzylamine and isoproterenol were prepared separately at a concentration of 1 mg/mL in 0.2 M perchloric acid at -80 °C.

### Animals

F344/NSlc rats (*n* = 15) (male, 8 weeks old, Japan SLC, Inc., Hamamatsu, Japan) were housed in groups of two and maintained on a 12-h light/dark cycle (lights on at 8 AM) with unlimited access to food and water. These rats were allowed to acclimate to their new environment for at least 1 week before being used in experiments. The animals used here were maintained and handled in accordance with the National Research Council’s Guide for the Care and Use of Laboratory Animals and our institutional guidelines.

### Neurotoxin treatments

Rats were treated twice with a neurotoxin one week apart to degenerate noradrenergic nerves and used for PET measurements 1–2 weeks after the last administration. First, rats were pretreated with the 5-HT reuptake blocker fluoxetine (10 mg/kg, i.p.) to protect serotonergic terminals. DSP-4 (5 mg/kg in 5 rats or 50 mg/kg in 6 rats, i.p., Sigma-Aldrich, Saint Louis, MO, USA) or saline in four rats (Otsuka Pharmaceutical Co., Ltd., Tokyo, Japan) was administered 30 min after pretreatment.

### Preparation of [^11^C]MRB

Radioactive ^11^C was generated by the ^14^N(p, α)^11^C nuclear reaction using a cyclotron. [^11^C]CH_3_I was prepared from [^11^C]CO_2_ according to the conventional method. The ^11^C methylation of (2*S*,3*S*)-Desethylreboxetine to [^11^C]MRB, HPLC purification and formulation were performed using an automated synthesis system (CUPID C-11-BII, Sumitomo Heavy Industries, Tokyo, Japan). Thus, obtained [^11^C]CH_3_I was trapped in 250 µL of anhydrous DMF containing 0.5 mg of (2*S*,3*S*)-Desethylreboxetine (1.75 µmol) and 8.5 µL of 5 M NaOH (42.5 µmol) at − 15 °C to − 20 °C, and then the reaction mixture was heated to 100 °C for 10 min. The radioactive mixture containing [^11^C]MRB was diluted with 1 mL of HPLC mobile phase and transferred to a column (10 mm I.D. × 250 mm, CAPCELL PAK C18, SHISEIDO, Tokyo, Japan) attached to an HPLC system (JACSO, Tokyo, Japan). Elution with 30:70 v/v CH_3_CN/0.2 M ammonium formate in sterile water at a flow rate of 5 mL/min yielded a radioactive fraction corresponding to pure [^11^C]MRB (retention time: 9.3 min). The fraction was collected in a rotary evaporator and evaporated to dryness at approximately 90 °C under reduced pressure. The residue was dissolved in 3 mL of sterile tween-saline and filtered through a 0.22 µm Millex^Ⓡ^-GV filter (Merck Millipore Ltd., USA). At the end of the synthesis, 0.5–1.3 GBq of [^11^C]MRB was obtained with a molar activity of 34–102 GBq/µmol.

### Brain PET scans

All rats (*n* = 15) (male, 10–12 weeks old, 226 ± 26 g) underwent [^11^C]MRB PET brain imaging. Rats were scanned for 60 min with our small animal PET scanner (FX3200, TriFoil Imaging, CA, USA) after injection of [^11^C]MRB (24.3–66.8 MBq, 0.34–4.1 nmol) via the tail vein under isoflurane anesthesia (~ 2.0%). All PET images were reconstructed using the three-dimensional ordered subset expectation maximization method (4 subsets and 20 iterations; voxel size: 0.6 × 0.5 × 0.5 mm with a resolution of 0.92 mm full width at half maximum at the center of the view). The time frames were 1 min × 10 frames, 2 min × 5 frames and 4 min × 10 frames.

### PET data analysis

Noradrenaline transporter densities in the hippocampus were estimated from dynamic PET data calculating the outcome measure, non-displaceable binding potential (*BP*_ND_). In brief, summed PET images (Frame #1–10) were manually coregistered with a rat brain MR template, then the same rigid transformation was applied to the corresponding dynamic PET images. Then, tentative *R*_1_ (relative blood flow) parametric images were estimated using MRTM2 with the globus pallidus as the reference region. Second, the tentative *R*_1_ images were manually coregistered with a rat brain MR template, then the revised rigid transformation was applied to the corresponding dynamic PET images to extract time-activity curves in the hippocampus and globus pallidus. Then, the multilinear reference tissue model 2 [17] was applied using the globus pallidus as the reference tissue (t* = 0.5 min, *k*_2_' = 0.02 min^−1^) to estimate *BP*_ND_.

We use the tentative *R*_1_ images because *R*_1_ reflects distribution of blood flow in the brain, allowing more accurate coregistarion by *R*_1_’s clearer outline of brain than does the summed early phase images. We chose the globus pallidus as the reference region based on the following reasons. First, the region was reported to express minimal amount of noradrenaline transporters in rat brain [18]. Second the region showed the lowest time-activity curves in our studies. Third, the region appeared not to be affected by spillover effect from the extra brain high radioactivity. The* k*_2_' value was calculated from the averaged *k*_2_' values estimated using Ichise’s multilinear reference tissue model [17] in the whole brain of four rats treated with 0 mg of DSP4. All PET data analyses were performed in PMOD 4.3 (PMOD Technologies LLC, Zurich, Switzerland).

### In vitro measurement of noradrenaline transporter levels

The protein levels of the norepinephrine transporter in the hippocampus of eight rats were determined by Western blotting. These samples were homogenized in RIPA buffer (FUJIFILM Wako Pure Chemical Corporation, Japan) with Protease and phosphatase inhibitor cocktail (cOmplete™ and PhosSTOP™; Roche Diagnostics GmbH, Germany). After centrifugation at 1000 × *g*, the protein levels of the supernatants were measured using the protein assay kit (TaKaRa BCA Protein Assay kit; Takara Bio, Inc., Japan). Samples containing 4 μg of protein were loaded onto 10% SDS–polyacrylamide gels for electrophoresis. Protein bands in the gels were transferred to polyvinylidene difluoride membranes by electroblotting. The membranes were incubated overnight at 4 °C with respective primary antibodies. These antibodies include a polyclonal antibody against rabbit noradrenaline transporter (3 μg/mL; Merck Millipore Ltd., USA) or a polyclonal antibody against mouse β-actin (1:5000 dilution; Proteintech, USA). The membranes were then further incubated with secondary antibodies (peroxidase-conjugated goat anti-rabbit IgG, 1:3000 dilution; Proteintech, USA, and peroxidase-conjugated goat anti-mouse IgG, 1:3000 dilution; Abcam, USA). Immunoreactive bands were visualized by enhanced chemiluminescence (Thermo Scientific, USA). Bands were detected using ImageQuant LAS-4000 (GE Healthcare, USA). Bands were evaluated by densitometry using ImageJ software (National Institute of Health, Bethesda, USA). OD values of noradrenaline transporter signals were compared and normalized to the maximum intensity in the control group.

### In vitro measurement of noradrenaline levels

The level of noradrenaline in the hippocampus of all the rats was measured by using an HPLC equipped with electrochemical detection (ECD). From the rat brains removed immediately after PET imaging, the hippocampi were sectioned and weighed, then frozen in liquid nitrogen and ground into powder using a grinding mill (SK-100, Tokken, Chiba, Japan). The ground samples were then homogenized in ice-cooled 0.2 M perchloric acid containing 0.2 µg/mL DHBA and IPT using an ultrasonic homogenizer (VP-050N, TAITEC, Tokyo, Japan). The suspensions were left on ice for 30 min, then centrifuged at 12,000 rpm for 15 min at 4 °C, and the supernatants were filtered through a 0.45 µm Cosmonice Filter W (Nacalai Tesque, Kyoto, Japan). Protein levels were measured using a BCA protein assay kit (Takara Bio Inc., Shiga, Japan). Monoamine levels in the samples and noradrenaline standard were measured using HPLC-ECD (ECD-700; Eicom, Kyoto, Japan) equipped with a reversed-phase column (EICOMPAK SC-50DS, 3.0 mm × 150 mm; Eicom, Kyoto, Japan) with mobile phase (3.10 g/L sodium acetate 8.84 g/L citric acid monohydrate, 220 mg/L SOS, 5 mg/L EDTA-2Na, and 20% methanol) pumped at a flow rate of 0.4 mL/min.

### Hematoxylin and eosin staining and immunohistochemistry

Brain sections including the pons were subjected to hematoxylin and eosin staining and immunohistochemistry of dopamine β-hydroxylase to confirm degeneration in the locus coeruleus by DSP-4 treatment. For hematoxylin and eosin staining, frozen tissue brain sections were stained with Mayer’s hematoxylin solution (FUJIFILM Wako Pure Chemical Corporation, Japan) for 5 min at r.t. and then rinsed with water. They were stained with Eosin Y (FUJIFILM Wako Pure Chemical Corporation, Japan) for 3 min at r.t. and then rinsed with water. They were dehydrated through an increasing gradient of ethanol, 70%, 90% and 99%, for 5 min each. They were cleared in xylene three times for 3 min each before mounting with Mount-Quick (COSMO BIO Co., LTD., Japan). Images were captured with a microscope (BZ-X800, Keyence, Japan).

For immunohistochemistry, frozen brain sections were immobilized with 10% formalin neutral buffer solution (FUJIFILM Wako Pure Chemical Corporation, Japan) at 4 °C for 1.5 h. They were then treated with 0.3% Triton-X in PBS, 1% BSA at r.t. for 1.0 h. They were incubated with Blocking One Histo (NACALAI TESQUE, INC., Japan) for 10 min at r.t. to block non-specific staining, followed by incubation in a chamber at 4 °C overnight with the primary antibody (anti-dopamine β-hydroxylase antibody, clone 4F10.2, MAB308, Merck, Germany). After washing with PBS-T, they were incubated with the secondary antibodies (goat anti-mouse IgG (H + L) highly cross-absorbed secondary antibody, Alexa Fluor 546, A-11030, Thermo Fisher SCIECTIFIC K.K., Japan) at r.t. for 1 h. They were covered with VECTASHIELD Hard Set Mounting Medium with DAPI (Vector Laboratories, Inc., CA, United States). Images were captured with a fluorescence microscope (BZ-X800, Keyence, Japan).

#### Statistics

Results are expressed as mean ± SD of replicate experiments. Multiple group comparisons were performed by one-way analysis of variance followed by Dunnett’s post hoc multiple comparison test. Statistical analyses were performed using Prism v9 software (GraphPad Software Inc., La Jolla, CA). Differences were considered significant at *p* < 0.05.

## Results

**Fig. 1:**
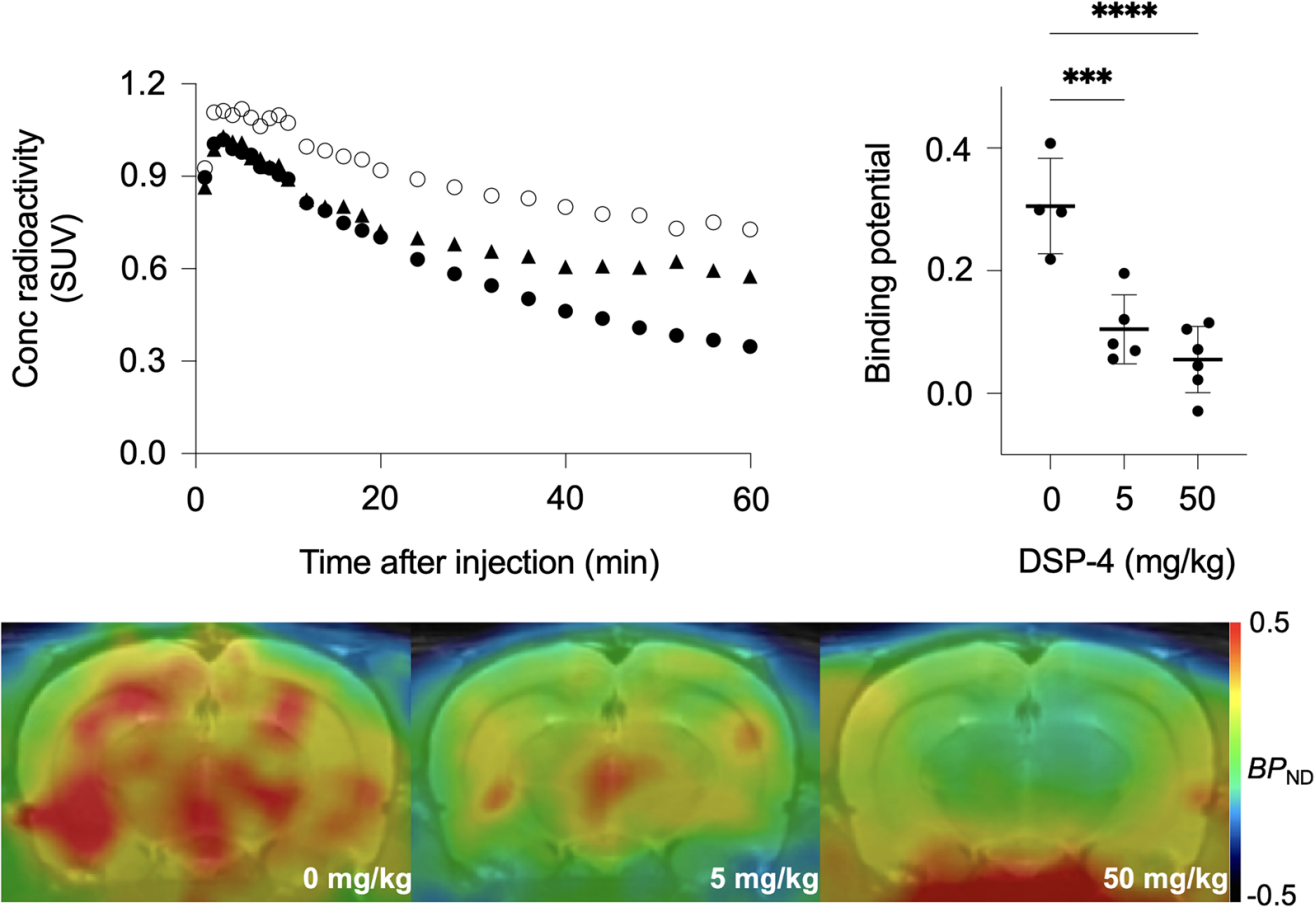
PET imaging of noradrenaline transporter densities in DSP-4 treated rats. *Top Left*: Time-activity curves of hippocampus of rats treated with 0 (*open circle*), 5 (*triangle*) and 50 mg/kg (*closed circle*) DSP-4. Data are average of four, five and six animals, respectively. *Top Right*: Binding potential in the hippocampus of rats treated with 0, 5 and 50 mg/kg DSP-4. Dunnett’s multiple comparison test showed significant differences between 0 and 50 (****: *p* < 0.0001) and 0 and 5 mg/kg DSP-4 (***: *p* < 0.001). *Bottom*: Averaged parametric images of rats treated with 0 (*n* = 4, *left*), 5 (*n* = 5, *middle*) and 50 mg/kg (*n* = 6, *right*) DSP-4. The coronal images in the hippocampus level were displayed with color scale units in non-displaceable binding potential (*BP*_ND_) overlayed on template MR images

[^11^C]MRB PET showed decreased noradrenaline transporter densities in a DSP-4 dose-dependent manner in the hippocampus of rats (one-way ANOVA, *F*(2, 12) = 20.98, *p* < 0.0001). The binding potentials of [^11^C]MRB PET were decreased by 66% and 82% in rats after DSP-4 administration at the dose of 5 and 50 mg/kg, respectively (0.31 ± 0.078 for 0 mg/kg, 0.10 ± 0.056 for 5 mg/kg, 0.055 ± 0.054 for 50 mg/kg DSP4, Fig. 1).

**Fig. 2:**
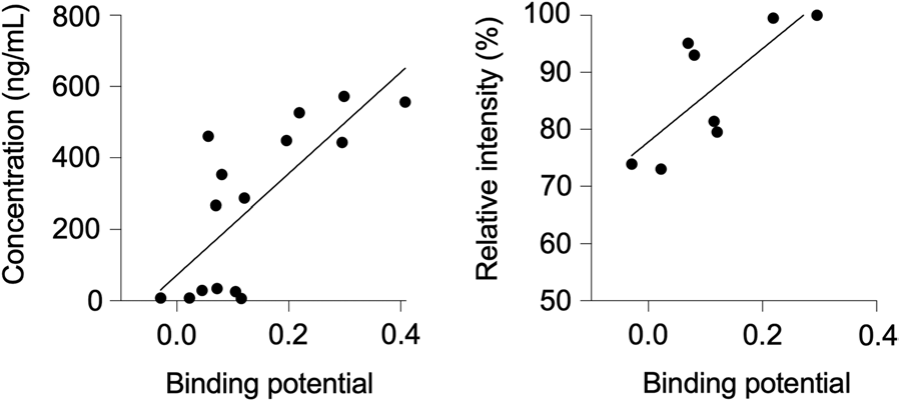
Correlations between PET measured noradrenaline transporter densities and in vitro measured noradrenaline concentrations as well as in vitro transporter densities. The binding potentials of [.^11^C]MRB PET correlated with noradrenaline concentration (*Left*) and the levels of noradrenaline transporter (*Right*)

The PET measured noradrenaline transporter densities correlated very well with in vitro measured noradrenaline concentrations as well as in vitro transporter densities. The binding potentials of [^11^C]MRB PET correlated with noradrenaline concentration (*R*^2^ = 0.57, Fig. 2A) and the levels of noradrenaline transporter (*R*^2^ = 0.58, Fig. 2B).

## Discussion

Our study shows that the decreased concentrations of noradrenaline transporters in the rat hippocampus measured by PET reflect decreased availability of noradrenaline in the same region. Noradrenaline transporter densities measured by [^11^C]MRB PET decreased in a DSP-4 dose-dependent manner in the hippocampus of DSP-4 treated rats. These PET measured noradrenaline transporter densities correlated very well with in vitro measured noradrenaline concentrations as well as in vitro transporter densities.

Our results suggest that [^11^C]MRB PET measurements of regional noradrenalin transporters are practically a good indirect index of regional noradrenaline concentrations. The 82% decrease in [^11^C]MRB binding potential in the hippocampus of rats treated with 50 mg/kg DSP-4 compared to those with 0 mg/kg is consistent with a previous study investigating cortical [^3^H]nisoxetine binding in DSP-4 treated rats [19]. With a single administration of 50 mg/kg DSP-4, [^3^H]nisoxetine binding decreased by 86% whereas noradrenaline concentration decreased by 100%.

Noradrenaline concentrations in the noradrenergic synaptic cleft are regulated by reuptake of noradrenaline through noradrenaline transporters; and at the same time, expression of noradrenaline transporters is regulated by extracellular noradrenaline. The relationship between the monoamine and monoamine transporter has been well-studied in the dopamine system. In dopamine homeostasis, dopamine transporters are downregulated when extracellular dopamine levels decrease [20]. Assuming that a similar mechanism to that of dopamine regulation holds for the noradrenergic system, the degree of noradrenaline transporter densities may overestimate the degree of loss of noradrenergic neurons due to downregulation of noradrenaline transporters in the face of decreasing number of intact noradrenergic neurons.

In our study, the noradrenaline transporter protein level decreased only by 30%, whereas the binding potential decreased by 82% in the hippocampus of rats treated with 50 mg/kg DSP-4 compared to those with 0 mg/kg. There are several possible explanations for this discrepancy between the above cited in vitro study and our in vivo PET study. In vivo, decreases in noradrenaline levels after lesioning of noradrenaline nerve terminals may lead to internalization of noradrenaline transporters located on the membrane of noradrenaline terminals resulting in more profound decreases in the transporter density measured by PET as opposed to much higher total protein levels including those in the cytoplasm by Western blotting.

Another possible explanation is that residual DSP-4 in the brain may interfere competitively or irreversible with binding of [^11^C]MRB to the noradrenaline transporters even one week after the final DSP-4 administration. It is unclear how DSP-4 damages the axonal terminals of noradrenergic neurons originating from the locus coeruleus [21]. While the primary toxic effect of DSP-4 is due to the accumulation of a reactive aziridinium derivative in the noradrenergic nerve terminals via the noradrenergic transporter, DSP-4 also acts as an irreversible inhibitor of the noradrenaline transporter. However, the irreversible binding site is not identical to that for noradrenaline transport, but is located in the inner part of the protein.

Lastly, the discrepancy may be related to the sensitivity of [^11^C]MRB PET in detecting small amounts of noradrenaline transporters. However, we believe that the sensitivity of PET measurements is not the issue, because our findings of decreased noradrenaline transporter as measured by PET was consistent with those of the previous study that employed a much more sensitive measurement technique. In that study using [^3^H]Nisoxetine, available binding sites decreased to a similar extent to ours after the treatment with DSP-4 [19].

In our study, we used DSP-4 to selectively lesion noradrenaline neurons in rats. The DSP-4 neurotoxin is highly selective for noradrenaline neuronal cells, but it can also harm serotonin neurons to a lesser degree [22, 23]. In the current study, we pre-administered fluoxetine to prevent DSP-4 from being taken up by serotonin neurons via the serotonin transporter. Although we did not quantify the number of noradrenaline neurons in the hippocampus, the loss of noradrenaline neurons was qualitatively confirmed by visually examining the neurons in the locus coeruleus (Additional file 1: Fig. S1). In addition, the intactness of the serotonergic system in this study was confirmed by checking the normal serotonin concentration (Additional file 1: Fig. S2).

We used HPLC-ECD to quantify noradrenaline concentration in the hippocampus. Noradrenaline was decreased by 31 and 96% in the hippocampus of rats administered 5 and 50 mg/kg DSP-4, respectively. This reduction was almost consistent with the previous studies showing ~ 10 and ~ 90% reduction in the hippocampus of rats one week after a single administration of 6.25 and 50 mg/kg DSP-4, respectively [24], and 35% and 82% reduction after 5 and 50 mg/kg DSP-4, respectively [25]. The magnitude of noradrenaline decrease with 5 mg/kg DSP-4 is similar to the decrease in the hippocampus of Alzheimer’s disease patients (~ 30%) [26]. Thus, [^11^C]MRB PET might be useful to detect decreased noradrenaline concentration due to neurodegeneration of locus coeruleus in Alzheimer’s disease patients.

Finally, our PET data analysis did not evaluate other cortical regions because high soft tissue spillover effects hamper accurate analysis of the cortex close to the skull considering the small size of rat brains with the limited spatial resolution of the PET system, although this situation might be of less issues in the human brain. However, the noradrenergic neurons are densely projected in the hippocampus, and PET signal was high enough here allowing us to perform data quantification analysis. The reference region used for the multilinear reference tissue model was the pallidum, which is an internal structure away from soft tissue and devoid of noradrenaline transporters.

A limitation of the current study is the limited accuracy of the *BP*_ND_ values of [^11^C]MRB PET. Identifiability of PET measured *BP*_ND_ values was modest to poor as *BP*_ND_ values became smaller (0.4–0.0) with 15 ± 2%, 33 ± 11% and 28 ± 58% standard error of estimates for rats treated with 0, 5 and 50 mg of DSP4, respectively. This relatively low PET measurement accuracy with our current radioligand is related to the fact that the noradrenaline transporter density is very low in the brain, which is approximately one tenth of the dopamine transporter density in the striatum [27]. Given this limitation of relatively low sensitivity of PET measurement of noradrenaline transporter densities, however, we found an excellent linearity between *BP*_ND_ and in vitro relative transporter intensity used as a gold standard here (*R*^2^ = 0.58), assuming that in vitro measurements are more accurate. This latter finding suggests that our *BP*_ND_ PET measurements are reliable within the given limitation of [^11^C]MRB.

One way to increase the sensitivity and accuracy of PET measurements of noradrenaline transporter densities is to use radioligands with a higher binding affinity for these transporters than does [^11^C]MRB. However, future efforts are needed to develop higher affinity radioligands for this transporter. Currently, (*S,S*)-[^18^F]FMeNER-D_2_ has been available, which has a slightly lower binding affinity compared with [^11^C]MRB (*K*_i_ 3.1 vs. 2.5 nM) [11]. However, [^18^F]FMeNER-D_2_ shows slightly superior sensitivity in detecting noradrenaline transporter densities in human cerebral cortex, probably due to higher signal-to-noise ratios with ^18^F-labeling [28].

In the current study in rats, [^11^C]MRB PET can detect 10–20% decreases in hippocampal noradrenaline transporter densities as a result of noradrenaline neuronal damage brought out by lesioning with 5 mg/kg DSP-4 treatment. In one human study, PET was sensitive to detect the decrease in the cortical availability of noradrenaline transporters using atomoxetine as a blocking agent, a noradrenaline reuptake inhibitor. However, in that study, PET failed to show a dose-dependent blocking agent effect on the PET measured transporter densities [12]. Thus, [^11^C]MRB may not be sensitive enough to detect small changes in noradrenaline transporter densities in brain of Alzheimer’s disease patients. For human studies, you can alternatively use (*S,S*)-[^18^F]FMeNER-D_2_, which has shown slightly superior sensitivity in detecting noradrenaline transporter densities in human cerebral cortex [28].

## Conclusions

[^11^C]MRB PET may be used as an in vivo biomarker of noradrenaline concentrations in the hippocampus of the neurodegenerating brain. Further studies appear warranted to extend its applicability to AD studies.

### Supplementary Information

**Additional file 1.** Supplementary figures.

## Data Availability

The datasets generated and/or analyzed during the current study are available from the corresponding author on reasonable request.
