# Supplementary material for: Noradrenaline transporter PET reflects neurotoxin-induced noradrenaline level decrease in the rat hippocampus
Source: EJNMMI Res. 2023 Sep 15;13:82. doi: 10.1186/s13550-023-01032-y (PMC10504202; doi:10.1186/s13550-023-01032-y)
Supplement: Supplementary file 1 — Additional file 1. Supplementary figures. [file 13550_2023_1032_MOESM1_ESM.docx]

Noradrenaline transporter PET reflects neurotoxin-induced noradrenaline level decrease in the rat hippocampus.

Takayuki Sakai^1^, Saori Hattori^1^, Aya Ogata^1,2^, Takashi Yamada^1^, Junichiro Abe^1^,
Hiroshi Ikenuma^1^, Masanori Ichise^1^, Masaaki Suzuki^1^,
Kengo Ito^1^, Takashi Kato^1^, Yasuyuki Kimura^1^*

Supplementary figures


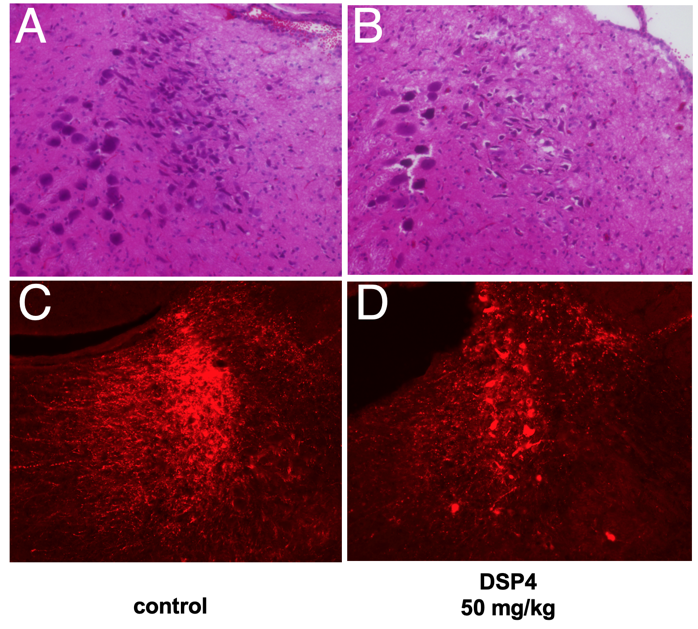


**Supplementary Figure 1.** The degeneration of noradrenaline neurons in the locus coeruleus. Hematoxylin and eosin staining (top) and dopamine β-hydroxylase immunohistochemistry (bottom) of the coronal sections including the locus coeruleus of the rats without (A&C) and with DSP-4 treatment (50 mg/kg, B&D).

**
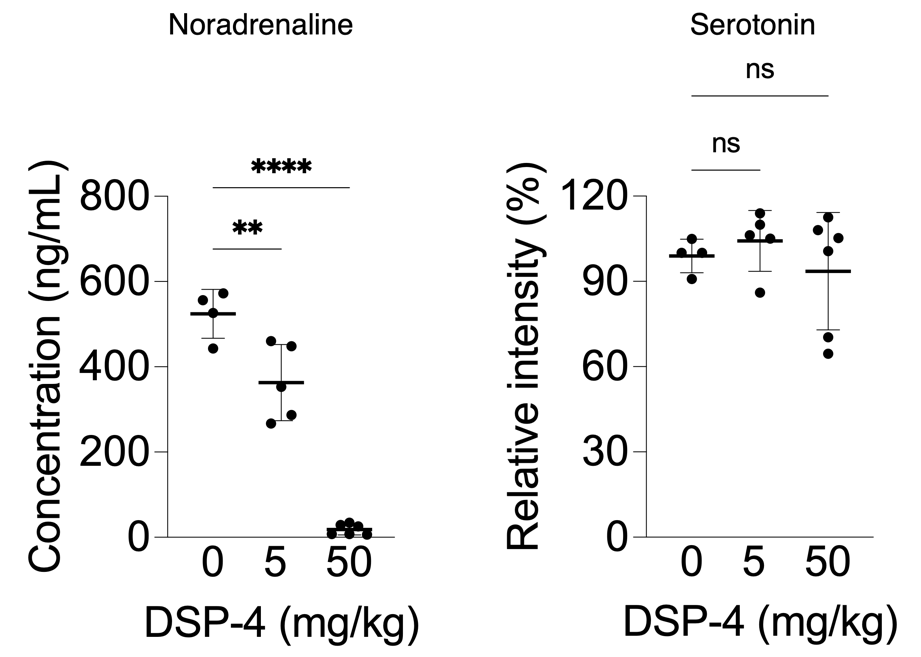
**

**Supplementary Figure 2.** Concentration of noradrenaline and serotonin measured with HPLC-ECD in the hippocampus of the rats with DSP-4 treatment (0, 5, 50 mg/kg).
